# Impact of Knee Pain on Fear of Falling, Changes in Instrumental Activities of Daily Living, and Falls Among Malaysians Age 55 Years and Above

**DOI:** 10.3389/fpubh.2020.571196

**Published:** 2020-10-14

**Authors:** Sumaiyah Mat, Shahrul Bahyah Kamaruzzaman, Ai-Vyrn Chin, Maw Pin Tan

**Affiliations:** ^1^Ageing and Age-Associated Disorders Research Group, Faculty of Medicine, University of Malaya, Kuala Lumpur, Malaysia; ^2^Geriatric Division, Department of Medicine, Faculty of Medicine, University of Malaya, Kuala Lumpur, Malaysia; ^3^Centre for Innovation in Medical Engineering, University of Malaya, Kuala Lumpur, Malaysia; ^4^Department of Medical Sciences, Faculty of Healthcare and Medical Sciences, Sunway University, Subang Jaya, Malaysia

**Keywords:** aged, knee pain, prospective falls, accidental falls, activity of daily living

## Abstract

**Objectives:** To determine the temporal relationship between the presence of knee pain and knee pain severity identified at baseline with fall risk, fear of falling and changes in instrumental activity of daily living at 12-months follow-up.

**Methods:** This was a prospective study from the Malaysian Elders Longitudinal Research (MELoR) study involving community dwelling older persons aged 55 years and older. The presence of one fall in the preceding 12 months, knee pain, and functional capacity were determined at baseline (2013–2015) and follow-up (2015–2016). Function was determined as loss of at least one of seven instrumental activities of daily living (IADL). Physical performance was evaluated at baseline using the timed-up-and-go (TUG) test. Fear of falling (FoF) was determined using the single question “Are you afraid of falling?”

**Results:** Data were available for 605 participants, mean (*SD*) age = 69.10 (7.24) years. Knee pain was present in 30.2% at baseline. Neither the presence of knee pain nor knee pain severity at baseline were associated with falls at 1-year follow-up. Knee pain was significantly associated with FoF at follow-up [aRR (95%CI) = 1.76 (1.02–3.04)] but not changes in IADL. Among individuals with no falls at baseline, the presence of knee pain was protective of falls at follow-up after adjustment for baseline physical performance [adjusted rate ratio, aRR (95% confidence interval, CI) = 0.35 (0.13–0.97)].

**Conclusion:** Knee pain is associated with increased FoF at 1.5 years' follow-up within a multi-ethnic population aged 55 years, residing in an urban location in a middle-income South East Asian nation. Interestingly, after differences in muscle strength was accounted for, knee pain was protective against falls at follow-up. Our findings challenge previous assumptions on joint pain and falls and highlights the importance of large prospective studies and further mechanistic research incorporating psychological factors in this area of increasing prominence.

## Introduction

Knee pain due to osteoarthritis (OA) is the most common type of joint pain complaint among older persons (1). The prevalence of knee pain, however, varies according to geographical location and culture with a higher prevalence reported among older Asians. One in three Malaysians age 55 years and above experience knee pain (2), while symptomatic knee OA has been reported in 16% of older individuals living the US (3). The presence of low grade, chronic pain in the knee due to OA, may negatively influence the older persons psychological state and overall quality of life.

Falls and fear-of-falling (FoF) are a debilitating conditions associated with poorer health status and functional decline among older adults (4–6). The consequences of falls includes fatal and non-fatal injuries (7), with serious injuries found in 20% of falls in older adults (8). In a recent systematic review, which included 39 studies, joint pain is associated with poorer static, dynamic, multicomponent, and reactive balance which may increase the risk of falls in older persons (9). The presence of balance impairment or instability resulting from joint pain may lead to FoF with the presence of actual fall events (10). It has been suggested that pain stimulates fear avoidance, (11) which leads to fear of movement and activity avoidance. The presence of knee pain may also lead to impairment in activities of daily living which may in turn lead to further increased FoF and other negative psychological sequelae (9).

Few studies have evaluated the relationship between knee pain with falls, FoF and functional status. The establishment of the temporal relationship between these key variables will informing the development of strategies to reduce the burden of disease associated with knee OA. Our previous work demonstrated a positive association between knee pain and retrospective recall of falls in the preceding year, in a cross-sectional analysis of 1,212 older Malaysian aged 55 years and above from the Malaysian Elderly Longitudinal Research (MELOR) cohort (12). With the subsequent availability of longitudinal follow-up data, we were able to examine the relationship between knee pain and falls in a time-dependent fashion. In addition, the effect of knee pain on FoF and changes functional status in measured with instrumental of activity daily living (IADL) were also studied.

## Methods

### Study Design and Population

First and second wave data were obtained from the Malaysian Elders Longitudinal Research (MELoR) study. The MELoR cohort was recruited between November 2013 to October 2015 from the electoral rolls of the Parliamentary constituencies of Petaling Jaya North, Petaling Jaya South, and Lembah Pantai which were located in Greater Kuala Lumpur. The recruitment strategies for MELoR have been explained in greater detail elsewhere (13). Individuals aged 55 years and above were selected through simple random sampling stratified by age deciles and ethnicity. Second wave data were obtained from 2015 to 2016. This study was approved by the University of Malaya Medical Centre Medical Ethics Committee (Ref: 925.4) and complied with the Helsinki Declaration of 1975, revised in 1983. Written informed consent was obtained from all study participants prior to their inclusion. Participants with communication difficulties, including cognitive impairment, affecting their ability to respond to the questionnaire were excluded.

### Baseline Interview

Participants were recruited through door-to-door visits. Data on demographics, socioeconomic started, home environment, media use, psychological status, functional status, falls history, medical history, medication use, healthcare utilization, and opinions on the end of life were obtained through computer-assisted interviews during this initial visit. Medical history was established through self-reported physical diagnosis of medical conditions identified on a list using commonly used terminology. The occurrence of falls was determined by asking participants during their home-based interviews whether they had at least one fall in the past 12 months. The presence of FoF was also established at baseline during this visit. This initial survey interview took around 2 h. The survey questionnaire was development through a series of face-to-face meetings and electronic communications by an expert panel comprising researchers in geriatrics, primary care, public health, economics, built environment, sports science, media studies, education, law, and computer science. Interviews were conducted by trained researchers blinded to the actual research questions. Participants were then requested to attend a hospital-based health check during which anthropometric and physical performance measurements were collected.

### Case Definition

The presence of pain was determined with the single question “Are you often troubled with pain?” Those who responded “yes” to the above question were then asked, “Do you have pain in any of the following parts of your body.” They were then required to select any appropriate responses from a list of seven options, which included: head, back, hip, knees, feet, mouth/teeth, and “all over.” With the assistance of the interviewer, participants then filled in a table requiring then to identify whether the pain affected the right, left, or both knees and to rate the severity of pain as “1 = mild,” “2 = moderate,” and “3 = severe.” This question was adopted from The Health and Retirement Study (HRS), a nationally representative survey of community-living older adults in the United States (14).

### Physical Performance

Physical performance in our participants was determined by hand grip strength (HGS) and the timed-up-and-go (TUG) test.

*Hand grip strength* was measured using a Jamar digital smart hand dynamometer (Pattersons Medical®/Samsons Preston®, USA). Participants' hand dominance was first determined. The researcher then first demonstrated the correct procedure to the participant, while instructing the participant to sit upright on a standard chair with back support and arms, and to start with the dominant arm. Participants were then instructed to hold their arms flexed at 90°, with the forearm resting on the arm of the chair, and to grip the dynamometer with their maximal strength. They were told they had three attempts with each arm. The researcher would then pass the instrument to the participant and instruct the participant to sit down on the chair and check the participant's position. Participants were encouraged to perform as well as they could using a standard squeezing phrase “Squeeze……harder, harder…and stop squeezing.” Three measurements (in kg) for each hand, alternating sides were recorded.

*The Timed Up and Go test* was first demonstrated to the participant, followed by one trial run, before taking a second measurement which was recorded. Shoes were kept on for this test. The time taken for the participant to complete a three-meter continuous walk from and back to a seated position on a standardized chair, 46 cm in height, with arms and a back rest was recorded. Participants were instructed to walk independently at their natural pace and were allowed to use a walking aid if they normally required one. Completion time of longer than 13.5 s (s) indicated impaired lower limb function (15).

### Follow-Up

Follow-up data were obtained during wave two interviews conducted between September 2015 to January 2016. Only home-based computer-assisted interviews were conducted during this second wave.

### Outcomes

#### Falls

Participants were again asked if they had the question, “Have you fallen in the last 12 months?” during the second wave. Those who provided an affirmative response were subsequently asked to report the number of falls they experienced.

#### Fear of Falling

Participants were asked, “Are you afraid of falling?” Those who answered “yes” to this question were considered to have FoF. Previous studies have found the single question to be comparable to falls efficacy scales in the determination of the presence of FoF in population-based studies (6, 16).

#### Changes in Instrumental Activities of Daily Living

Seven out of eight items of the Lawton-Brody IADL were included in the home-based interview questions (ability to use telephone, going out, shopping, food preparation, doing housework, taking own medication, and ability to handle finances). The item on managing own laundry as it was felt by the expert panel that it did not appropriately measure activities of daily living in the Malaysian culture where tasks are commonly delegated to younger female in the family regardless of the older person's ability to perform such tasks. A score of “1” was assigned to each item if they responded positively to “answering phone calls,” “using transportation with assistance,” “shopping independently,” “preparing meals independently,” “doing housework with help,” “managing their own medications,” and “managing everyday finances with help with banking and major transactions,” or “0” if they rated their function were below the above stipulated levels for each item (17). The maximal score was therefore “7,” with a lower score indicating poorer function. Changes in IADL (cIADL) at follow-up were determined by subtracting the wave two IADL score from the wave one IADL score (cIADL = IADL_baseline_ – IADL_follow−up_). The score was then dichotomised with those with a score of one or greater categorized as reduced IADL and 0 and below as no change or improved IADL.

### Statistical Analysis

**Figure 1:**
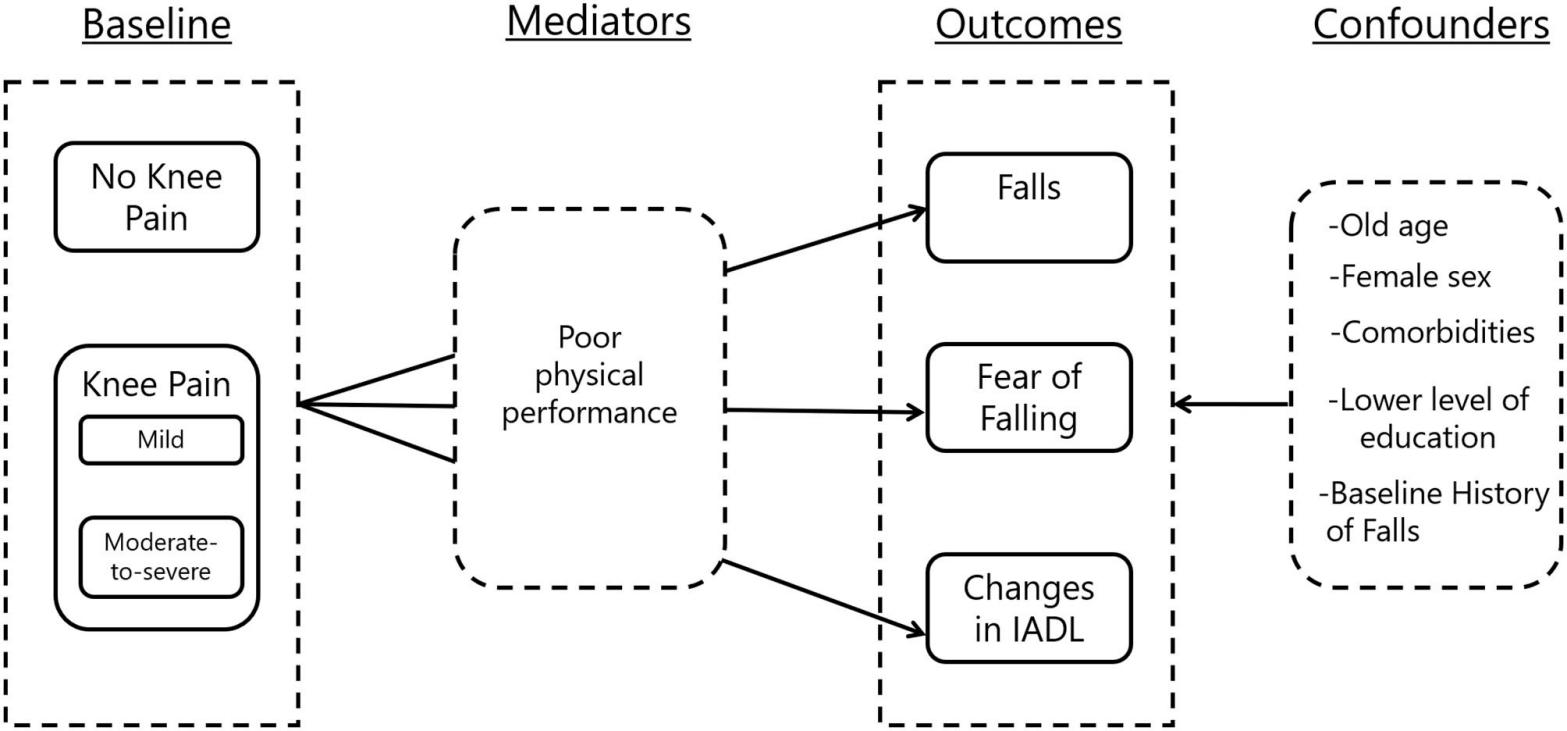
Theoretical framework on the relationship between knee pain, falls, fear of falling, and changes in instrumental activity of daily living. IADL, instrumental activities of daily living.

Data analyses were conducted using the SPSS Version 20 (IBM, Armonk, NY, USA). Descriptive statistics were first presented as means with standard deviations for continuous data and frequencies with percentages for categorical data. The independent *t*-test was applied for continuous variables and chi-squared test for nominal variables in bivariate analyses. Subsequently, the rate ratios (RR) with 95% confidence intervals (CI) were determined for falls at wave 2, for all participants and the sub-group of non-fallers at baseline. In addition, similar comparisons were made for severity of knee pain using logistic regression with dummy variables. Uncertain responses and missing values were removed in the association analysis. Multiple logistic regressions analyses were performed to assess the association between knee pain as well as knee pain severity and falls following adjustments for demographic differences and baseline physical performance. We first included falls in the year prior to baseline as a covariate in multivariable models. As the presence of falls at baseline was a strong predictor of subsequent falls, a subgroup comprising only non-fallers as baseline was created by excluding those with baseline falls. Analyses were repeated by substituting fear of falling and reduced IADL as the outcomes. Baseline IADL was not included as covariate in the multivariable analysis in order to avoid multicollinearity effects. Potential confounders were selected based on differences in baseline characteristics and clinical relevance. The theoretical framework by which this analysis strategy was drawn is further illustrated in Figure 1.

## Results

### Baseline Characteristics

The initial recruitment figures for the MELoR cohort comprised 1,614 individuals at wave one home-based computer assisted interviews. Subsequently 1,419 attended hospital-based health checks. Wave two interviews were abruptly stopped after 770 participants were re-interviewed due to withdrawal of study funding in 2016 resulting from sudden, catastrophic economic circumstances. Data on knee pain, falls, physical performance and follow-up falls were available for a total of 605 (79.5%) participants, mean (SD) age 69.10 (7.24) years. The mean time to follow-up between wave one and wave two was 494.1 days. Table 1 displays participant characteristics at baseline according to knee pain status. Participants with knee pain were more likely to be women with lower educational attainment, and increased likelihood of self-reported physician-diagnosed hypertension and diabetes mellitus. Individuals with knee pain also had higher body mass index (BMI), poorer physical performance measured by TUG and HGS and lower IADL scores. Individuals with knee pain were also more likely to have reported the occurrence of at least one fall in the preceding 12 months and FoF at enrolment to the study (Table 1).

**Table 1:** Baseline characteristic for participants with and without knee pain.

	**Without knee pain (*n* = 422)**	**With knee pain (*n* = 183)**	***p*-value**
Age (*y*), mean (*SD*)	68.90 (7.00)	69.55 (7.76)	0.312
**Sex**, ***n*** **(%)**			
Female	211 (50.0)	133 (72.7)	<0.001^*^
**Education Level**, ***n*** **(%)**
Primary and lower	66 (15.7)	62 (33.9)	<0.001^*^
**Comorbidities**, ***n*** **(%)**			
Hypertension	197 (46.7)	107 (58.5)	0.008^*^
Diabetes mellitus	102 (24.2)	60 (32.8)	0.028^*^
Stroke	8 (1.9)	2 (1.1)	0.477
Heart attack	27 (6.4)	17 (9.3)	0.208
Asthma	30 (7.1)	11 (6.0)	0.622
Parkinsonism	2 (0.5)	0 (0.0)	0.351
Visual problem	163 (38.6)	83 (45.4)	0.122
Ischemic heart disease	62 (14.7)	17 (9.3)	0.070
BMI (kg/m^2^), *mean (SD)*	24.81 (4.35)	26.28 (4.56)	<0.001^*^
Medication ≥5, *n* (%)	158 (37.5)	82 (45.1)	0.083
TUG score, *mean (SD)*	11.73 (2.95)	13.39 (4.74)	<0.001^*^
Grip strength, *mean (SD)*	24.59 (7.97)	20.70 (6.64)	<0.001^*^
IADL score, *mean (SD)*	6.68 (0.76)	6.45 (0.99)	0.002^*^
Fear of falling, *n* (%)	290 (68.9)	158 (86.8)	<0.001^*^
History of falls, *n* (%)	84 (19.9)	62 (33.9)	<0.001^*^

*SD, Standard deviation; BMI, Body mass index; TUG, timed-up-and-go; IADL, instrumental activities of daily living*.

* *p < 0.05*.

### Knee Pain and Prospective Falls

The association between knee pain and prospective falls was determined for the overall cohort as well as those who had no falls at baseline according to the presence of knee pain and severity of knee pain. In other words, logistic regression analyses were conducted for all participants, as well as non-fallers at baseline (no falls in the past 12 months at wave 1). Prospective falls (falls in past 12 months at wave 2) was considered the dependent variable while knee pain or moderate or severe knee pain were independent variables. Table 2 documents the crude and adjusted associations between knee pain and knee pain severity with falls in all subjects and subjects without falls at baseline. The presence of knee pain or moderate to severe knee pain at baseline was not associated with prospective falls at follow-up. In the sub-group analysis, those without history of falls showed similar results where the presence of knee pain was not associated with prospective falls. However, following adjustment for baseline physical performance which either HGS or TUG score, presence of knee pain or moderate-to-severe knee pain were protective of prospective falls (adjusted rate ratio, aRR = 0.35; 95% confidence interval, CI = 0.13–0.97). This suggests the presence of knee pain protected individuals who had no previous history of falls from any subsequent falls once differences in sociodemographic, comorbidities, and physical performance or muscle strength were accounted for statistically (Table 2).

**Table 2:** Associations between baseline knee pain and prospective falls.

**Prospective Falls, *n* (%)**	**Knee pain**		**Knee pain severity**		
	**No knee pain**	**With knee pain**	**No pain**	**Mild**	**Moderate-to-severe**
**All participants (*****N*** **=** **605)**					
Unadjusted RR (95% CI)	1	1.03 (0.67–1.60)	1	0.88 (0.41–1.88)	1.01 (0.61–1.67)
Adjusted RR (95% CI)^a^	1	0.91 (0.57–1.48)	1	0.88 (0.40–1.94)	0.85 (0.49–1.48)
Adjusted RR (95% CI)^b^	1	0.84 (0.51–1.36)	1	0.80 (0.36–1.77)	0.78 (0.44–1.37)
Adjusted RR (95% CI)^c^	1	0.65 (0.89–0.97)	1	0.66 (0.28–1.54)	0.56 (0.30–1.02)
**Baseline no falls (*****N*** **=** **459)**					
Unadjusted RR (95% CI)	1	0.56 (0.27–1.14)	1	0.55 (0.16–1.85)	0.55 (0.16–1.85)
Adjusted RR (95% CI)^a^	1	0.50 (0.23–1.09)	1	0.52 (0.15–1.80)	0.40 (0.15–1.08)
Adjusted RR (95% CI)^b^	1	0.45 (0.20–0.99)^*^	1	0.49 (0.14–1.71)	0.35 (0.13–0.97)^*^

*RR, rate ratio; CI, confidence interval*.

a *Model adjusted with age, sex, education, hypertension, diabetes and BMI*.

b *Model further adjusted with muscle strength (grip strength), adjustment for TUG produced similar result*.

c *Model further adjusted with baseline history of falls*.

* *Significant at p < 0.05*.

### Knee Pain and Fear-Of-Falling at Follow-Up

Table 3 summarizes the multiple logistic regression analyses using the presence of FoF at wave 2 follow-up as the dependent variable, with either the presence of knee pain or knee pain severity as independent variables. Analyses were conducted first with the overall cohort then with individuals with no falls at baseline (participants with at least one fall in preceding 12 months at recruitment excluded). Both crude unadjusted RR with 95% CI as well as aRR with 95%CI after adjustment first for age, sex, education, hypertension, diabetes, and BMI, followed by further adjustments with HGS are presented here. Unadjusted analyses found significant associations between presence of knee pain and FoF at follow-up for the overall cohort (RR = 2.66; 95% CI = 1.62–4.36) as well as baseline the non-faller (RR = 3.36; 95% CI = 1.28–6.26) subgroup. For the overall cohort the presence of knee pain remains significantly associated with FoF at follow-up after the first adjustment for potential confounders (aRR = 1.76; 95% CI = 1.02–3.04), but the relationship was no longer significant following additional adjustment for HGS (aRR = 1.71; 95% CI = 0.99–2.98). In the non-fallers at baseline subpopulation, the relationship between presence of knee pain and FoF remained significant after adjustment for all potential known confounders. When knee pain severity was considered, crude and adjusted analyses for identical confounders and potential mediators found that individuals with moderate to severe baseline knee pain were significantly more likely to have FoF at follow-up than those without baseline knee pain for the overall cohort as well as the baseline non-faller subgroup (Table 3).

**Table 3:** Presence and severity of knee pain at baseline and fear of falling at follow-up.

**Fear of falling**	**Knee pain**		**Knee pain severity**		
	**No knee pain**	**With knee pain**	**No pain**	**Mild**	**Moderate-to-severe**
**All participants (*****N*** **=** **605)**					
Crude RR (95% CI)	1	2.66 (1.62–4.36)^*^	1	1.75 (0.83–3.70)	3.24 (1.75–5.97)^**^
Adjusted RR (95% CI)^a^	1	1.76 (1.02–3.04)^*^	1	1.20 (0.54–2.67)	2.21 (1.12–4.37)^*^
Adjusted RR (95% CI)^a^	1	1.71 (0.99–2.98)	1	1.16 (0.52–2.60)	2.12 (1.07–4.19)^*^
Adjusted RR (95% CI)^c^	1	1.69 (0.97–2.94)	1	1.14 (0.51–2.56)	2.07 (1.04–4.10)^*^
**Baseline no falls (*****N*** **=** **459)**					
Crude RR (95% CI)	1	3.36 (1.81–6.26)^**^	1	2.62 (0.99–6.90)	3.78 (1.76–8.13)^*^
Adjusted RR (95% CI)^a^	1	2.53 (1.28–5.03)^**^	1	1.82 (0.66–5.03)	3.04 (1.29–7.20)^*^
Adjusted RR (95% CI)^a^	1	2.52 (1.26–5.03)^**^	1	1.83 (0.66–5.07)	3.02 (1.27–7.19)^*^

*RR, rate ratio; CI, confidence interval*.

a *Model adjusted with age, sex, education, hypertension, diabetes and BMI*.

b *Model further adjusted with muscle strength (grip strength), adjustment for TUG score produced similar result*.

c *Model further adjusted with baseline history of falls*.

* *Significant at p < 0.05*.

** *Significant at p < 0.01*.

### Knee Pain and Change in Instrumental Activities of Daily Living

Table 4 summarizes the logistic regression analysis findings using cIADL as the dependent variable and either presence of knee pain or knee pain severity as independent variable. In the unadjusted analysis, having knee pain or moderate-to-severe knee pain was associated with reduced IADL (crude RR = 2.00; 95% CI = 1.29–3.11). This association was, however, attenuated following adjustment of age, comorbidities, BMI, and demographic differences. Similar findings were observed when we compared mild knee pain and moderate-to-severe knee pain with absence of knee pain. Among baseline non-fallers, no significant association was found in all models (Table 4).

**Table 4:** Knee pain and knee pain severity at baseline and reduction in instrumental activities of daily living.

**Reduction in IADL**	**Knee pain**		**Knee pain severity**		
	**No knee pain**	**With knee pain**	**No pain**	**Mild**	**Moderate-to-severe**
**All participants (*****N*** **=** **605)**					
Crude RR (95% CI)	1	2.00 (1.29–3.11)^*^	1	2.08 (1.05–4.12)^*^	2.03 (1.24–3.32)^**^
Adjusted RR (95% CI)^a^	1	1.62 (0.98–2.68)	1	1.83 (0.86–3.89)	1.59 (0.90–2.80)
Adjusted RR (95% CI)^b^	1	1.65 (0.99–2.74)	1	1.85 (0.87–3.96)	1.62 (0.92–2.87)
Adjusted RR (95% CI)^c^	1	1.65 (0.98–2.78)	1	1.89 (0.87–3.97)	1.64 (0.92–2.91)
**Baseline no falls (*****N*** **=** **459)**					
Crude RR (95% CI)	1	1.91 (1.11–3.28)	1	2.18 (0.97–4.94)	1.83 (0.98–3.42)
Adjusted RR (95% CI)^a^	1	1.45 (0.79–2.65)	1	1.90 (0.80–4.53)	1.26 (0.62–2.58)
Adjusted RR (95% CI)^b^	1	1.46 (0.79–2.69)	1	1.91 (0.80–4.55)	1.27 (0.62–2.62)

*RR, rateratio;CI, confidenceinterval*.

a Model adjusted with age, sex, education, Hypertension, diabetes and BMI.

b *Model further adjusted with physical performance (Grip strength), adjustment for TUG score produced similar result*.

c *Model further adjusted with baseline history of falls*.

* *Significant at p < 0.05*.

** *Significant at p < 0.01*.

## Discussion

The temporal relationships between knee pain and falls, fear of falling and changes in function are evaluated in this study. By examining the prospective relationship between the presence of knee pain and the severity of knee pain with falls at follow-up, FoF at follow-up and cIADL, we were able to tease out the potential influence of knee pain on physical, psychological and functional outcomes in residents aged 55 years at recruitment in an urban location in Malaysia, a multi-ethnic, upper-middle income country in South-East Asia. The presence of knee pain at baseline is not associated with increased risk of falling at one-and-a-half-year follow-up but was conversely protective of follow-up falls once those who fell at baseline were excluded once differences in muscle strength were adjusted for. The presence of knee pain at baseline, was, however, associated with the presence of FoF at follow-up. Changes in IADL over the follow-up period associated with baseline knee pain was found attributable to differences in age, sex, level of education and comorbidities.

Few studies have obtained data on fall occurrence prospectively in relationship to knee pain (18). A study by Dore et al. suggested that the presence of lower limb OA in one site led to a 53% increase the risk of future falls in 12 months (19). In contrast, our finding was more in line with a recent study by the European Project on OSteoArthritis (EPOSA) which found that the presence of clinically-diagnosed knee OA, was not associated with one or more falls at 1-year follow up. Instead, this study found significant associations between clinical OA and recurrent falls with pain medication as potential mediators (20). Our study, therefore, complements the findings of the EPOSA study by further determining whether FoF or functional status was affected prospectively by the presence and severity of knee pain.

As neither the presence of knee pain or knee pain severity led to falls over 12 months over a mean follow-up duration of 1.5 years, we went on explore this relationship using statistical methods, in an attempt to explain this unexpected finding, despite the expected presence of poorer muscle strength, gait and balance scores and IADL performance among those with knee pain. Individuals who had reported at least one fall in the preceding 12 months at enrolment were excluded from our exploratory analyses since a previous history of falls is a strong risk factor for subsequent falls (21–23) and may have confounded our findings. Following adjustments for differences in comorbidities, age, gender, education and muscle strength or gait and balance between those with and without knee pain, the presence of knee pain appeared to confer a protective effect on falls occurrence at follow up. This protective effect appeared to apply in those with moderate to severe knee pain over those with no knee pain. While such exploratory analyses ought to be interpreted with caution, it has revealed a potential mechanistic explanation for why the presence of knee pain may not necessarily lead to falls. It is to be expected that knee pain is associated with increasing age, female gender, reduced muscle strength and increased BMI which are associated with increased risk of falls (24), the effect of pain may conversely balance out the increased risk from the above through a mechanism which remains unclear. Plausible explanations may include, increased vigilance, compensatory strategies and reduced physical activity (25, 26). This could have important implications on treatment strategies, which have thus far concentrated primary on pain relief. Removal of the sensation of pain may paradoxically lead to increased risk of falls, as highlighted by the EPOSA study.

Despite the lack of association of knee pain with fall occurrence at follow-up, FoF appeared to be independently associated with presence of knee pain, with the relationship relevant for those with moderate to severe knee pain rather than those with mild knee pain. The relationship was unchanged after baseline fallers were excluded. The implication of FoF is not explained in this study as FoF was considered an outcome, and the relationship between FoF and falls and other potential physical and psychological sequelae was not explored. Previous studies have considered FoF as a psychological condition with greater negative consequences than falls alone, with established relationships between FoF and activity avoidance and impaired functional status (4, 27).

While significant associations between knee pain at baseline and deterioration in IADL was found initially, this was confounded by advanced age, increased BMI, comorbidities, and demographic differences. Knee pain did not lead to any change in IADL among baseline non-fallers. A previous study by the Osteoarthritis Initiative showed over 7 years' follow-up, out of 1,055 adult aged 45 to 79 years, with or at high risk of knee OA who had no limitation at baseline, 25% had slow and steady decline and another 5% had fast and progressive decline (28). Thus, it is possible that, the real impact of having knee pain on IADL can only be demonstrated over a longer follow-up duration.

### Limitations

Fall occurrence at follow-up was dependent on retrospective recall. However, given the resource limitations for following up this cohort, which also led to incomplete follow-up of the cohort, prospective diary exercises were not within our grasp. Potential recall bias may, therefore, lead to inaccuracies in our findings, and which may have influenced the lack of any significant relationship between knee pain and falls in our cohort. Funding issues commonly affect cohort studies, which is also a major reason why cohort studies from developing countries remain limited. The presence of knee pain was only identified through the lead-in question, “are you often troubled by pain.” The duration of the pain had not been provided here with the word “often” being a subjective term, therefore further adding to the potential inaccuracies. No confirmatory diagnosis of osteoarthritis with clinical assessments or radiographical methods was possible. Our study nevertheless has highlighted the need to conduct further studies which may further unwrap the potential protective effect of pain on fall occurrence as well as to examine psychological and economic implications, which this study was unable to address as it addressed psychological, functional and fall outcomes concurrently, and did not examine the interplay between these three outcomes.

This study highlighted the complexity of the relationship between knee pain with falls and other fall related outcomes. By concurrently examining the effect of knee pain on falls, fear of falling and functional capacity prospectively, we found that knee pain at baseline did not increase the risk of falls in the preceding 12 months over a mean follow-up period of 1.5 years. The relationship between knee pain and FoF was, however, apparent, but not changes in functional ability. Future studies should consider evaluating the potential protective effect of knee pain which could be due to increased vigilance and activity avoidance from fear of falling.

## Data Availability Statement

Due to concerns about loss of fidelity of personal identifiable data the MELoR data set is currently not available publicly. However, parts of the data set will be release anonymized through written requests submitted to the corresponding author.

## Ethics Statement

The studies involving human participants were reviewed and approved by University of Malaya Medical Centre Medical Ethics Committee (Ref: 925.4). The patients/participants provided their written informed consent to participate in this study.

## Author Contributions

SM, SK, and MT: conceptualization, investigation, and methodology. SM and MT: formal analysis. SK, A-VC, and MT: funding acquisition. All authors: contributed to the article and approved the submitted version.

## Conflict of Interest

The authors declare that the research was conducted in the absence of any commercial or financial relationships that could be construed as a potential conflict of interest.
